# All for One and One for All: Voluntary Physicians in the Intensive Medicine Units During the COVID-19 Outbreak in Spain

**DOI:** 10.1017/dmp.2020.375

**Published:** 2020-10-12

**Authors:** Teresa Nunez-Villaveiran, Alejandro González-Castro, Emilio Nevado-Losada, Abelardo García-de-Lorenzo, Pau Garro

**Affiliations:** Plastic and Reconstructive Surgery Unit, Hospital General de Granollers, Barcelona, Spain; Intensive Care Department, Hospital Universitario Marques de Valdecilla, Santander, Spain; Intensive Care Department, Hospital Universitario Príncipe de Asturias, Madrid, Spain; Intensive Care Department, Hospital Universitario La Paz, Madrid, Spain; Intensive Care Department, Hospital General de Granollers, Barcelona, Spain

**Keywords:** COVID-19, disaster medicine, hospital organization, intensive care medicine, mass critical care, surge capacity

## Abstract

**Objectives:**

Our purpose was to determine the intensive care units’ (ICU’s) medical staff surge capacity during the coronavirus disease 2019 (COVID-19) outbreak in Spring 2020 in Spain.

**Methods:**

A multicenter retrospective survey was performed addressing the medical specialties present in the ICUs and the increase in bed capacity during this period.

**Results:**

Sixty-seven centers (62.04%) answered the questionnaire. The ICU bed capacity during the pandemic outbreak increased by 160% (95% confidence interval [CI], 128.97-191.03%). The average number of beds per intensive care medicine (ICM) specialist was 1.5 ± 0.60 and 3.71 ± 2.44 beds/specialist before and during the COVID-19 outbreak, respectively. Non-ICM specialists and residents were present in 50 (74.63%) and 23 (34.3%) ICUs during the outbreak, respectively. The number of physicians (ICM and non-ICM residents and specialists) in the ICU increased by 89.40% (95% CI, 64.26114.53%). The increase in ICM specialists was, however, 4.94% (95% CI, −1.35-11.23%). Most non-ICM physicians were anesthetists, followed by pediatricians and cardiologists.

**Conclusions:**

The majority of ICUs in our study were able to rapidly expand critical care capacity by adapting areas outside of the normal ICU to manage critically ill patients, and by extending the critical care staff with noncritical care physicians working as force multipliers.

A new viral pneumonia caused by severe acute respiratory syndrome coronavirus 2 (SARS-CoV2) was first detected in December 2019 in Wuhan, China. The outbreak extended rapidly to the rest of the world and was declared a pandemic on March 11, 2020.^1^ The new coronavirus disease 2019 (COVID-19) overwhelmed the health systems of different countries all over the world, including Spain.^2^ Until then, little was known about how staff would respond to catastrophic events such as naturally occurring virulent infectious disease outbreaks.^3^ Many hospitals faced a sudden increase in admissions of critical and noncritical patients, with a significant burden on their health-care infrastructure and workforce. This ongoing stream of new critically ill patients required individual hospitals to increase their intensive care unit (ICU) capacity. Furthermore, the demands on the ICU were prolonged, because critically ill patients required hospital stays that lasted for weeks.^4^ Numerous ICUs overflowed and required expansion of critical care beyond the ICU, in nonroutine ICU areas, with the support of physicians who were not intensivists.^5^

Our goal was to describe the ICU medical staff surge capacity during the COVID-19 pandemic in Spain. In particular, we wanted to establish the increase in ICU bed capacity and physicians (including critical and noncritical care specialists and residents) involved in the daily management of patients admitted to the adult ICUs during the outbreak of COVID-19 (March 1, 2020 to May 1, 2020) in Spain.

## METHODS

This study was a multicenter retrospective survey addressing the medical specialties present in the ICU during the Spring 2020 peak of COVID-19 in Spain. Participants included department chairs and attending physicians from different adult ICUs in Spain.

A 14-item survey was designed in Google Forms (Google LLC, Mountain view, CA; https://docs.google.com/forms) to address the following: (1) demographics, (2) number of ICU beds and intensive care medicine (ICM) specialists available before and during the pandemic outbreak, and (3) number non-ICM specialists easing the workload in the ICU during the pandemic outbreak (Table 1). The Hospital de Granollers Review Board reviewed the study procedures, and a written consent exemption was granted for the survey.

**TABLE 1:** Survey Questions

Question	Hospital Name
1	What was the number of beds in your ICU before the COVID-19 outbreak?
2	What has been the maximum number of ICU beds (including converted beds in other hospital floors) during the COVID-19 outbreak?
3	What was the number of ICM specialists in your ICU before the COVID-19 outbreak?
4	What was the number of ICM residents in your ICU before the COVID-19 outbreak?
5	During the COVID-19 outbreak, how many ICM specialists have been working in your unit?
6	During the COVID-19 outbreak, how many ICM residents have been working in your unit?
7	During the COVID-19 outbreak, how many non-intensive care specialists have been working in your unit?
8	Please describe the number of non-ICM specialists that have been working in your unit during the COVID-19 outbreak, as well as their specialties (eg, 10 anesthetists, 4 pediatricians, 2 general surgeons, 1 vascular surgeons, etc.). If someone has multiple specialties, please indicate the specialty that this person usually works in.
9	During the COVID-19 outbreak, how many non-intensive care residents have been working in your unit?
10	Can you describe the specialties in which these residents are training? (eg, 3 pediatricians, 2 general surgeons, etc.).
11	Did any of the specialists have a second specialty? If so, could you please indicate which were the specialties (eg, general surgery and internal medicine, pediatrics and anesthesia, etc.). Also include residents with a previous specialty.
12	Finally, would you like to make any comments regarding the responsibilities and roles of the other specialists, as well as your opinion regarding their help?

A link to the online survey was distributed during the first week of May 2020 by the 5 authors of this study to different intensivists in Spain directly by email and SMS messages, and to 2 groups of intensivists from Cataluña and Madrid using the WhatsApp (Facebook Inc., Menlo Park, CA) messaging application. The study was closed on May 10, 2020. This was a voluntary survey with no incentives offered. To prevent multiple entries, only 1 survey per hospital was used. If they were duplicated, we directly contacted the author to determine the correct survey. Only completed questionnaires were analyzed.

The population of each Autonomous Community by January 1, 2019, the accumulated incidence of COVID-19 cases per 100,000 persons by region on May 10, 2020, and the excess mortality (percentage difference between the observed count of deaths from all causes of death during a specific period, and the expected numbers of deaths in the same period) between March 17 and May 10, 2020, by region of Spain were also collected. These data are publicly available on the National Institute of Statistics webpage (https://www.ine.es), the Spanish Health Ministry webpage (https://www.mscbs.gob.es), and the Carlos III Institute webpage (https://www.isciii.es).

Statistical analyses were completed using SPSS Version 24.0 (IBM Corp, Armonk, NY). Tests were 2-tailed and values of *P* < 0.05 were considered statistically significant. A descriptive analysis of the sample was performed using frequencies and percentages, and average values and standard deviation (mean ± standard deviation). Continuous variables before and during the COVID-19 outbreak, such as the number of beds or physicians in the ICU, were compared using a paired t-test. A paired samples Wilcoxon test was used when the distribution was not normal. The Spearman correlation test was used to determine the association between the percentage increase (difference between maximum value during the COVID-19 outbreak and the value before the COVID-19 outbreak, expressed as a percentage of the value before the COVID-19 outbreak) in ICU capacity during the COVID-19 outbreak, and the percentage increase in total physicians, total specialists, ICM specialists, total residents, and ICM residents during the same period.

## RESULTS

A total of 67 centers answered the questionnaire. The response rate was 62.04%. Most centers were located in Barcelona and Madrid, 2 of the main cities in Spain. However, we were able to obtain data from all the Autonomous Communities in Spain except for La Rioja (Table 2). Most centers (82.1%) were public or mixed (public and private). Forty-one (61.2%) centers had ICM residents. The characteristics of the centers that answered the questionnaire are summarized in Table 3.

**TABLE 2:** Centers With ICU per Region, Centers That Participated in the Study, and Outbreak Cumulative Incidence and Excess Mortality per Region

Region (Ceuta and Melilla Are Excluded From the Analysis)	Number of Centers per Region That Answered the Questionnaire (%)	Number of ICUs Managed by ICM Specialists per Region^20^	Inhabitants (INE Data January 1, 2019, Consulted on www.ine.es)	Cumulative Incidence per 100,000 Population on May 10, 2020	Excess Mortality Between March 17 and May 10, 2020 (%)
Andalusia	5 (7.5%)	42	8,414,240 (17.9%)	6.9	17.5%
Aragon	1 (1.5%)	6	1,319,291 (2.8%)	27.4	70.6%
Asturias	2 (3.0%)	6	1,022,800 (2.2%)	7.9	28.2%
Balearic Islands	2 (3.0%)	6	1,149,460 (2.4%)	8.4	14.1%
Basque Country	1 (1.5%)	12	2,207,776 (4.7%)	31.8	75.2%
Canary Islands	1 (1.5%)	11	2,153,389 (4.6%)	4.2	5.0%
Cantabria	1 (1.5%)	5	581,078 (1.2%)	27.7	45.0%
Castile and Leon	2 (3.0%)	9	2,399,548 (5.1%)	70.6	111.3%
Castile - La Mancha	1 (1.5%)	9	2,032,863 (4.3%)	36.1	179.1%
Catalonia	18 (26.9%)	47	7,675,217 (16.3%)	68.7	70.3%
Extremadura	1 (1.5%)	5	1,067,710 (2.3%)	16.5	72.3%
Galicia	2 (3.0%)	14	2,699,499 (5.7%)	32.3	19.5%
La Rioja	0 (0%)	1	216,798 (0.7%)	46.4	78.8%
Madrid	24 (35.8%)	48	6,663,394 (14.2%)	52.0	173.8%
Murcia	1 (1.5%)	7	1,493,898 (3.2%)	2.3	13.4%
Navarre	1 (1.5%)	3	654,214 (1.4%)	52.0	135.0%
Valencian Community	4 (6,0%)	27	5,003,769 (10.6%)	11.1	28.3%

**TABLE 3:** Demographic Characteristics of the ICUs

Type of center (public or mixed vs private)	Public or mixed Private	55 (82.09%) 12 (17.91%)
ICU beds per center before the COVID-19 outbreak	<10 beds 10-19 beds 20-29 beds 30-39 beds 40-49 beds Not answered	19 24 9 12 2 1
Center with an ICM residency program	Yes No Not answered	41 25 1

**FIGURE 1:**
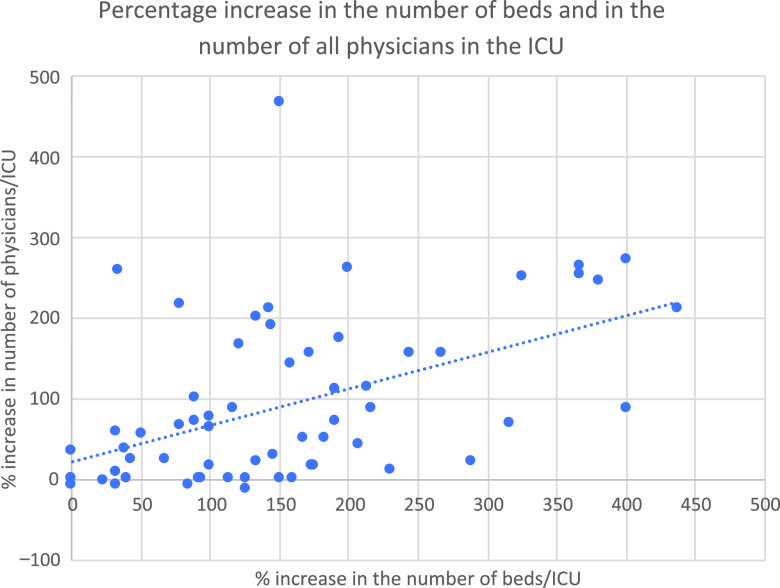
Percentage Increase in Number of Beds and Number of Physicians (ICM and non-ICM Residents and Physicians) in the ICU During the COVID-19 Outbreak.

The average pre-COVID-19 outbreak ICU beds before the pandemic were 17.76 ± 10.83 beds per center. The average ICU beds during the COVID-19 outbreak increased to 43.29 ± 28.40 beds per center. There was a percentage increase in bed capacity of 160 (95% confidence interval [CI], 128.97-191.03%) during the pandemic outbreak (Figure 1). The average number of beds per ICM specialist was 1.5 ± 0.60 beds/specialist before the standard deviation -19 outbreak, and 3.71 ± 2.44 beds/specialist during the outbreak. The percentage increase in physicians (ICM and non-ICM residents and specialists) in the ICU during the outbreak was 89.40% (95% CI, 64.26-114.53%). The percentage increase in ICM specialists, however, was of 4.94% (95% CI, -1.35-11.23%). The percentage increase in ICM and non-ICM residents during the outbreak was of 87.62% (95% CI, 40.08-135.16%). The percentage increase in ICM residents was 31.75% (95% CI, -1.71-65.21). These results are summarized in Table 4.

**TABLE 4:** Increase in ICU Bed Capacity and Physicians During the COVID-19 Outbreak (March-May 2020)

	Before the COVID-19 Outbreak Average (SD)	During the COVID-19 Outbreak Average (SD)	% Increase During COVID-19 Outbreak (IC95%)	*P*-Value
ICU beds per center	17.76 (10.83)	43.29 (28.40)	160.00% (128.97,191.03)	<0.001
Beds per ICM specialist	1.50 (0.60)	3.71 (2.44)	157.72 (120.44, 195.00)	<0.001
Beds per ICM residents (*n* = 41 centers)	6.33 (6.30)	11.16 (9.00)	139.88 (100.73, 179.02)	<0.001
Total physicians in the ICU	15.63 (10.36)	26.82 (18.54)	89.40% (64.26, 114.53)	<0,001
Total specialists (ICM and non-ICM) in the ICU	12.11 (6.37)	21.42 (12.56)	94.30% (67.53, 121.07)	<0.001
Total ICM specialists in the ICU	12.11 (6.37)	12.72 (7.15)	4.94% (-1.35, 11.23)	0.56
Total residents (ICM and non-ICM) in the ICU (*n* = 47 centers)	5.17 (4.61)	8.40 (8.55)	87.62% (40.08, 135.16)	<0.001
Total ICM residents in the ICU (*n* = 41 centers)	6.00 (4.38)	6.68 (4.24)	31.75% (-1.71, 65.21))	0.12

Fifty (74.63%) units received noncritical specialists’ help during the outbreak. Non-ICM residents were present in 23 (34.3%) units. Seven of these units had no ICM residents. One of them did not provide data on the number of them or their specialty. There was a moderate correlation between the percentage increase in critical care beds and the percentage increase in total physicians (ICM and non-ICM specialists and residents) (ρ = 0.521; *P* = 0.00001). The correlation between the percentage increase in critical care beds and percentage increase in ICM and non-ICM specialist physicians was the strongest (ρ = 0.529; *P* ≤ 0.00001). However, despite being significant, the correlation between the percentage increase in critical care beds and percentage increase in ICM specialist physicians was weaker (ρ = 0.296; *P* = 0.018). The correlation between the percentage increase in critical care beds and the percentage increase in total and in ICM residents was also moderate (ρ = 0.476; *P* = 0.002; and ρ = 0.353; *P* = 0.023, respectively).

**FIGURE 2:**
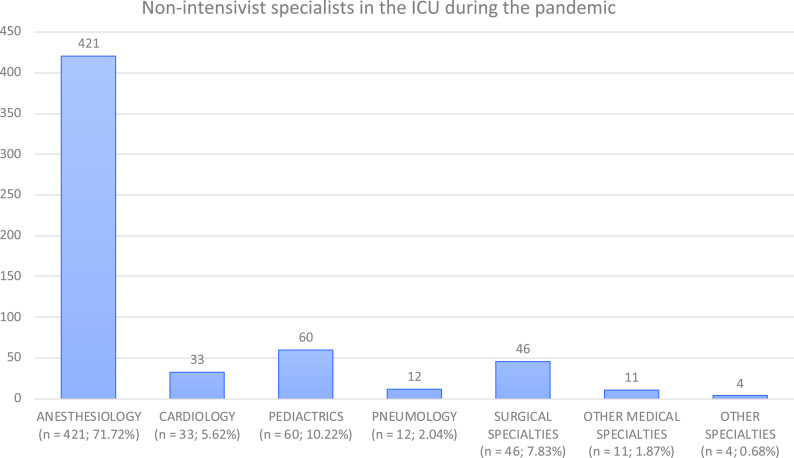
Nonintensivist Physician Specialists in the ICU During the COVID-19 Outbreak.

**FIGURE 3:**
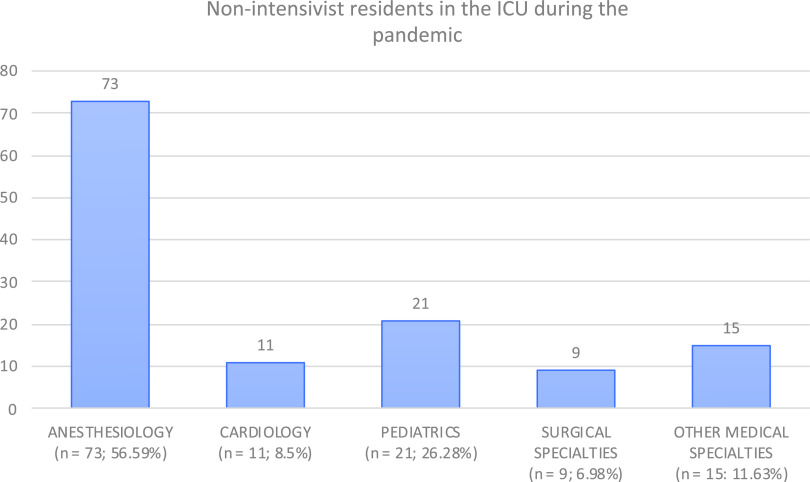
Nonintensivist Physician Residents in the ICU During the COVID-19 Outbreak.

The majority of non-ICM specialists were anesthesiologists (71.7%), followed by pediatricians (10.2%) and cardiologists (5.6%). A similar tendency was observed in the non-ICM resident pool, with the majority of them being anesthesia (56.6%), pediatrics (26.3%), and cardiology residents (8.5%) (Figures 2 and 3). Fifteen (2.6%) physicians had a double specialty, generally intensive care medicine and anesthesia.

## DISCUSSION

Surge capacity in mass critical care is the ICU’s ability to rapidly expand care to as many patients as possible in a disaster. An important component of surge capacity is the staff.^6^ A contagious epidemic, such as COVID-19, produces an ongoing stream of new patients that may go on indefinitely. In this setting, the number of critically ill patients rapidly overwhelms the capacity to care for them and may require any individual hospital to double or triple its ICU capacity.^7^ Furthermore, the demands on the ICU are prolonged because it is the end destination for critically ill patients with hospital stays that last for weeks.^4^ In addition, health-care workers in the ICU are at risk of acquiring the infection and potentially becoming critically ill themselves.^7^ Consequently, surge planning needs to be able to account for a prolonged duration of ICU functioning at a given surge capacity.^4^

In our study, the ICUs increased their bed capacity by an average of 160%, with an increase of 400% in some centers. Medical staff surge capacity depended on both critical care and noncritical care physicians. ICM specialists recruited to increase the staff during the pandemic were either former residents from the departments, physicians who were previously hired to take calls and/or were working as first responders, or retired physicians. In some cases, senior residents in their last year of training assumed the role of specialists. It was more difficult to hire intensivists in smaller units because of the competition for them with bigger hospitals, and their reduced residency positions.

The critical care staff was extended with noncritical care physicians working as “force multipliers.” A 2-tiered staffing system was established to provide care to more critically ill patients, with critical care staff (intensivists and critical care nurses) supervising noncritical care staff.^6-8^ There was an average increase in the number of physicians present inside the ICUs of nearly 90%, including non-ICM specialists. This allowed for an increase of beds managed by each intensivist (from 1 to 2 patients to 3 to 4 patients per ICM specialist). Patients were also managed by pneumologists using noninvasive mechanical ventilation in intermediate care units.

Each center organized their shifts. One of our centers organized 12-h work shifts with 2 intensivists and 1 anesthesiologist during the night shifts, and the rest of the team during the day shifts. Each physician did 3-d shifts and a 1-night shift every 5 d, with the remaining time of the week to rest. Another center increased the number of physicians on call (from 3 to 4 physicians) and, during the worse moments of the pandemic, added 2 physicians supporting the call team until 8 pm and 10 pm, respectively. Some centers were able to maintain their usual 8-8 shifts due to the support of the pediatric intensivists and the anesthesiologists. Others combined work shifts of 8 and 12 h, depending on the number of patients inside the ICU.

Most of the non-ICM specialist help was provided by anesthesiologists, followed by pediatricians and cardiologists. A possible explanation for this is that these 3 specialties manage their own ICUs (postoperative, pediatric-neonatology ICUs, and coronary ICUs, respectively). Furthermore, anesthesia specialists are ICU specialists in some centers. Generally, anesthesia either cooperated with ICM specialists in the care of COVID-19 critical patients inside the ICU or handled COVID-19 patients inside their own units independently. Another likely reason for the presence of pediatricians in the adult ICUs is that SARS-CoV-2 affects children less commonly and severely in comparison to adults.^9^ Pediatricians, in particular pediatric intensivists, were not as likely to be as overwhelmed as adult intensivists during the pandemic. In fact, in our survey, 1 center described that 10 beds inside the pediatric ICU were used for adult COVID-19 critical patients.

In the ICUs of the authors of this study, most of the help with the direct management of the patients was provided by anesthesiologists, and occasionally also by pediatric intensive care specialists, under the supervision of intensivists. Most intensivists managed 3 or 4 patients and supervised residents and other specialists, generally anesthesiologists who were seeing another 3 patients themselves. However, at a given point, some intensivists were taking care of 6 patients each.

The roles were determined depending on the previous training and experience of the volunteers present in the ICU. Non-ICM specialists were not always involved in the direct management of patients. For example, in some centers ENT specialists performed tracheostomies; orthopedic surgeons, gynecologists, and general surgeons made prone teams (teams to turn the patients onto their front to improve their oxygenation)^10^; pediatricians helped with note-writing and asked for labs, and psychiatrists gave information to the families. In addition to the direct care of patients, anesthesiologists also helped with airway management. Nevertheless, they occasionally lacked specific training in intensive care pathologies. The majority of the other specialists had to be supervised by the ICM specialists, in particular with the respiratory management of the patients.

Physicians face limitations when called for to assist in mass casualty situations without the benefit of formal education in disaster medicine.^11,12^ Currently, there is no standardized disaster curriculum for health-care professionals to create unified disaster medicine core competencies.^13^ An education strategy to prepare noncritical care physicians to assist in disasters as ICU physician extenders would be useful.^6,8^ Noncritical care staff would benefit from a basic adult and/or pediatric critical care course, providing basic concepts essential for critical care, such as recognizing critical illness, airway management, ventilator management, antimicrobial therapy, management of shock, neurocritical care, and basic management of trauma patient.^6^ Examples of these courses include the Fundamentals of Critical Care Support and the Fundamentals of Disaster Management.^7^ Education for nonpediatric critical care providers to manage critically ill children and vice versa and education for pediatric intensivists to comanage critically ill adults with adult intensivists should also be considered.^8^

The mortality rate in the ICU of the authors of this study usually ranges between 10 and 13%. During the pandemic, the mortality rate of COVID-19 patients was higher and varied between 25 and 35%. However, when we compare these rates with the mortality rates for acute respiratory distress syndrome (between 35 and 40%),^14^ the increase in ICU capacity did not seem to play a major impact on the outcomes.

Studies to determine willingness to work in disaster scenarios have found that the 3 most important factors influencing disaster response are concern for the safety of the family, belief in the physician’s duty to provide care, and availability of personal protective equipment (PPE).^15^ The ability and willingness have been reported to be lowest for fatal infectious agents and radiation events.^3,16^ However, in our study, most of the centers kept their usual number of ICM specialists and residents, and in some cases, they hired more specialists.

During the epidemic of SARS that started in 2002, 40% of the people who became infected in Toronto were health-care workers, many of them critical care professionals.^7^ During the 2014-2016 West African epidemic of Ebola Virus Disease (EVD), health-care workers in West Africa were shown to be at 21-32 times higher risk of contracting the disease compared with the general population, and by the end of the outbreak, 881 health workers had become infected and 513 had died from the virus.^18^ At a time when the demand for ICU support was increasing, the available staff was decreasing.^7^ Similarly, some respondents described that their ICUs had a shortage of their ICM specialists because they were infected by SARS-CoV2. Strategies such as health-care worker education, provision of adequate PPE, availability of proper treatment (or prophylaxis) for various dangerous agents, and assurance of effective environmental control are necessary.^3,11,16^

Our study has several limitations. First, because it is survey-based, it is subject to response bias and individual willingness to report. Second, the results may not be generalized to other health-care systems in other countries. In Spain, Intensive Care Medicine is a recognized medical specialty with a 5-y duration residency.^19^ Most critical adult patient beds (71%) depend upon the departments of ICM, and the rest are distributed in variable numbers among different specialties (particularly postoperative, coronary, and cardiac surgery units).^20^ The nursing personnel, which may or may not have specific training in critical care medicine, are also an important part of the staff of the services of ICM.^19^ However, we do not have advanced nursing providers, such as nurse practitioners and nurse anesthetists, or other providers, such as physician assistants and critical care paramedics. Moreover, we did not study what happened in centers in which the ICUs are managed solely by anesthetists and other specialists.

A third limitation is that most of the responses came from hospitals located in Madrid and Catalonia, with less participation of centers from other regions. This occurred because our survey was distributed by means of WhatsApp groups of physicians who came mainly from these 2 communities. Furthermore, larger hospitals from Madrid answered our survey, but this did not happen with most of the large hospitals in Catalonia. The reason why some of the referral hospitals in Catalonia did not answer our questionnaire is unknown. A possibility is that there was a drag effect within the WhatsApp group in Madrid because the chiefs of the bigger hospitals commented inside the chat group that they had answered the questionnaire. On the contrary, smaller hospitals in Catalonia were probably easier to organize; therefore, it was easier for their chiefs to know the data to answer directly to the questionnaire. Fourth, we did not study the gender, age, or marital status and number of children of the physicians who came to the ICU. In some studies, studying willingness to respond to disasters caused by biological agents, for example, men were significantly more likely to respond than women (67.9% vs 38.3%).^16^ Physicians may have childcare and eldercare obligations that might affect their ability to report to work in several ways.^3^ Finally, we have only studied the physician response in the ICU. In all of our units, nurses and other clinical support staff from other hospital areas worked in the newly improvised ICUs. Moreover, numerous physician specialists and residents supported the internal medicine department in the management of less severely affected COVID-19 patients and we did not include them in the study. Other medical and surgical specialty demographics were likely present in that setting.

## CONCLUSIONS

In our study, the COVID -19 outbreak overflowed the ICUs in different regions in Spain, with an average increase in their bed capacity of 160%. The majority of ICUs were able to rapidly expand critical care capacity by extending the critical care staff with noncritical care physicians working as force multipliers. In this 2-tiered staffing, ICM specialists supervised the other physicians, who were mostly anesthetists, pediatricians, and cardiologists. The ICUs should develop a disaster plan with the steps that need to be implemented to increase critical care resources to accommodate a large number of patients.

## Availability of Data and Material

Available upon request.
